# Paternal age and neonatal health: unraveling epigenetic pathways

**DOI:** 10.1093/hropen/hoaf052

**Published:** 2025-09-01

**Authors:** Yuquan Yuan, Chengzhi Zhao

**Affiliations:** Chongqing Health Center for Women and Children, Chongqing, China; Women and Children’s Hospital of Chongqing Medical University, Chongqing, China; Chongqing Health Center for Women and Children, Chongqing, China; Women and Children’s Hospital of Chongqing Medical University, Chongqing, China

Dear Editor,

We commend Xiong *et al.* (2025) for their investigation into the impact of paternal age on neonatal outcomes, particularly regarding its association with preterm birth and cesarean section. This study adds valuable insight into the role of paternal age in offspring health. However, several key issues warrant further consideration, particularly regarding biological mechanisms, maternal–paternal age interactions, and long-term effects on offspring health.

First, although the authors identified paternal age as a risk factor for neonatal outcomes such as preterm birth and cesarean section, they did not explore the biological mechanisms behind these associations. Advanced paternal age is linked to genetic mutations and epigenetic changes in sperm, but these mechanisms were not addressed. How do genetic mutations and epigenetic alterations in sperm contribute to preterm birth and other complications? Is it possible to understand the full impact of paternal age without exploring these biological pathways? Sperm quality declines with age, leading to DNA fragmentation, which has been associated with poor pregnancy outcomes (Stenqvist *et al.*, 2025). Moreover, telomere length in sperm decreases with age and is linked to early pregnancy loss and chromosomal abnormalities in offspring (Cariati *et al.*, 2016). The increase in epigenetic modifications in sperm with age may also alter gene expression in the embryo, influencing fetal development and leading to adverse outcomes. How can we confidently attribute negative neonatal outcomes solely to genetic mutations without considering epigenetic changes? These findings suggest that further investigation into sperm DNA fragmentation and epigenetic changes due to paternal age is crucial for understanding the full impact of paternal age on neonatal health.

Second, Xiong *et al.* (2025) highlight paternal age’s independent effects on neonatal outcomes but do not explore how maternal and paternal age interact to influence these outcomes. Given the complex interaction between maternal and paternal contributions to pregnancy, understanding how paternal age interacts with maternal age is vital. Why does the study overlook the combined effect of both parental ages, especially when each has a significant influence on neonatal health? Research has shown that advanced paternal age, when coupled with maternal age, increases the likelihood of birth defects and preterm birth (Halvaei *et al.*, 2020). Is it not essential to understand whether the combined effect of both parental ages leads to greater risks than the sum of their individual contributions? Additionally, both maternal and paternal age influence the quality of sperm and oocytes, with the combined effect exceeding individual contributions. The study should have examined this synergistic effect. Understanding how maternal and paternal age together influence neonatal outcomes is critical for identifying high-risk pregnancies and developing effective clinical interventions.

Third, although Xiong *et al.* (2025) evaluate neonatal outcomes, such as preterm birth and cesarean section, they fail to address the long-term consequences of paternal age on offspring health. As life course epidemiology gains prominence, it is essential to investigate how paternal age affects not only neonatal health but also childhood development, academic performance, and long-term health outcomes. Research has linked advanced paternal age with neurodevelopmental disorders such as autism spectrum disorder (ASD) and schizophrenia, which may manifest later in life. One study found that older paternal age is associated with a higher incidence of ASD in children, suggesting that genetic mutations related to paternal age could emerge later (Reichenberg *et al.*, 2006). If neonatal outcomes alone are studied, can we ignore the potential long-term neurodevelopmental impacts of paternal age? Children of older fathers are also at higher risk of cardiovascular and metabolic conditions later in life, highlighting the intergenerational effects of paternal age. How much of the long-term health risks in offspring are linked to paternal age-related genetic changes? Without considering these long-term consequences, how can the findings on neonatal outcomes fully reflect the broader implications of paternal age?

To conclude, while Xiong *et al.* (2025) contribute valuable insights into the relationship between paternal age and neonatal outcomes, critical questions remain. The lack of a biological mechanism to explain the observed effects, insufficient exploration of the interaction between maternal and paternal age, and the absence of longitudinal data on long-term health consequences limit the study’s comprehensiveness. We recommend that future studies delve deeper into the genetic, epigenetic, and environmental factors that influence the effects of paternal age on offspring health and include longitudinal studies to assess the full scope of its impact on neonatal and child development.
